# Social Stress Engages Neurochemically-Distinct Afferents to the Rat Locus Coeruleus Depending on Coping Strategy^1^,^2^,^3^

**DOI:** 10.1523/ENEURO.0042-15.2015

**Published:** 2015-11-26

**Authors:** Beverly A. S. Reyes, Gerard Zitnik, Celia Foster, Elisabeth J. Van Bockstaele, Rita J. Valentino

**Affiliations:** 1 Department of Pharmacology and Physiology, College of Medicine, Drexel University, Philadelphia, Pennsylvania 19102; 2 Department of Anesthesiology and Critical Care Medicine, The Children’s Hospital of Philadelphia, Philadelphia, Pennsylvania 19104

**Keywords:** c-fos, corticotropin-releasing factor, enkephalin, Fluorogold, locus coeruleus, resident–intruder

## Abstract

Stress increases vulnerability to psychiatric disorders, partly by affecting brain monoamine systems, such as the locus coeruleus (LC)-norepinephrine system. During stress, LC activity is coregulated by corticotropin-releasing factor (CRF) and endogenous opioids. This study identified neural circuitry that regulates LC activity of intruder rats during the resident–intruder model of social stress. LC afferents were retrogradely labeled with Fluorogold (FG) and rats were subjected to one or five daily exposures to an aggressive resident. Sections through the nucleus paragigantocellularis (PGi) and central amygdalar nucleus (CNA), major sources of enkephalin (ENK) and CRF LC afferents, respectively, were immunocytochemically processed to detect c-fos, FG, and CRF or ENK. In response to a single exposure, intruder rats assumed defeat with a relatively short latency (SL). LC neurons, PGI-ENK LC afferents, and CNA-CRF LC afferents were activated in these rats as indicated by increased c-fos expression. With repeated stress, rats exhibited either a SL or long latency (LL) to defeat and these strategies were associated with distinct patterns of neuronal activation. In SL rats, LC neurons were activated, as were CNA-CRF LC afferents but not PGI-ENK LC afferents. LL rats had an opposite pattern, maintaining activation of PGi-ENK LC afferents but not CNA-CRF LC afferents or LC neurons. Together, these results indicate that the establishment of different coping strategies to social stress is associated with changes in the circuitry that regulates activity of the brain norepinephrine system. This may underlie differential vulnerability to the consequences of social stress that characterize these different coping strategies.

## Abstract (precis)

Stress increases vulnerability to psychiatric disorders, partly by affecting brain monoamine systems, such as the locus coeruleus (LC)-norepinephrine system. During stress, LC activity is coregulated by corticotropin-releasing factor (CRF) and endogenous opioids.

## Visual Abstract

**Figure:**
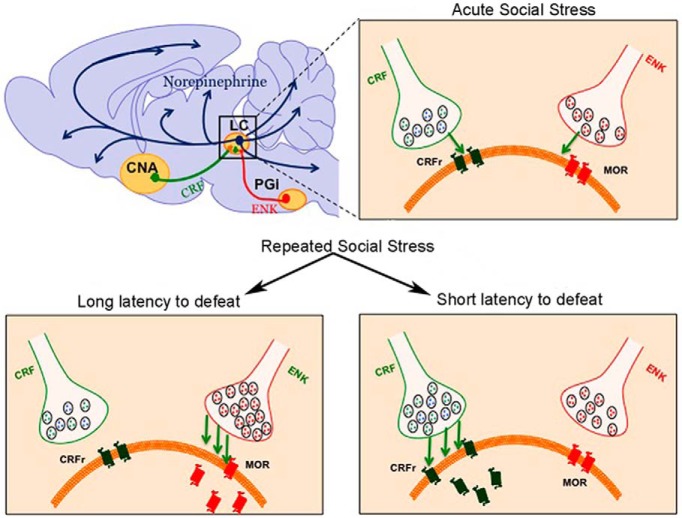

## Significance Statement

Social stress has been linked to psychiatric disorders, in part through activation of the locus coeruleus (LC)-norepinephrine system. This study identified circuits that are engaged during acute and repeated social stress to regulate this system. It was found that the establishment of different coping strategies with repeated social stress was associated with distinctions in stress-activated circuitry. In rats that resisted defeat, inhibitory enkephalin afferents to the LC were engaged, whereas in rats that are biased toward subordination, excitatory corticotropin-releasing factor inputs to the LC were engaged. The engagement of different circuits with opposing actions may underlie distinctions in the consequences of social stress in subjects with different coping strategies.

## Introduction

Successful adaptation to stressors requires the coordination of multiple stress response systems that mount adaptive endocrine, autonomic, immunologic, and behavioral responses. Whereas the immediate activation of these systems in response to acute stress is adaptive, continuous or chronic activation can have pathologic consequences (McEwen, 1998; de Kloet et al., 2005). The locus coeruleus-norepinephrine (LC-NE) system is a major brain stress response system. Activation of the LC-NE system is important in maintaining arousal and cognitive flexibility in response to acute stress (Berridge and Waterhouse, 2003; Valentino and Van Bockstaele, 2008). However, repeated or prolonged activation of this system has been implicated in arousal-related symptoms of stress-related psychiatric disorders, including depression and post-traumatic stress disorder (Chappell et al., 1986; Southwick et al., 1999; Wong et al., 2000). Identifying the neuromediators and circuitry that regulate LC activity during repeated stress is integral to our understanding of the arousal and cognitive domains of stress-related psychiatric diseases.

LC dendrites receive convergent synaptic input from separate axon terminals containing the stress-related neuropeptide, corticotropin-releasing factor (CRF), and enkephalin (ENK) (Tjoumakaris et al., 2003). CRF and ENK afferents to the LC derive from different nuclei, with the central nucleus of the amygdala (CNA) being a primary source of CRF and the medullary nucleus paragigantocellularis (PGi) being a primary source of ENK (Drolet et al., 1992; Van Bockstaele et al., 1998; Tjoumakaris et al., 2003). CRF and ENK have opposing excitatory and inhibitory effects on LC neuronal activity, respectively, and these are both engaged during acute stress (Valentino and Van Bockstaele, 2008, 2015). Although the predominant influence on LC activity during acute stress is CRF-induced excitation, antagonizing CRF reveals an underlying opioid inhibition that is thought to restrain LC activation and facilitate recovery to baseline activity after stressor termination (Curtis et al., 2001; Valentino and Van Bockstaele, 2008; Curtis et al., 2012).

For humans, stressors of a social nature are common and this can be modeled by the rodent resident–intruder stress in which an “intruder” rat is placed into the cage of a “resident” rat, which is typically larger and sufficiently aggressive, such that an attack on the intruder ensues (Koolhaas et al., 1997). Repeated resident–intruder stress produces hypothalamic-pituitary-adrenal axis dysfunction, decreased social interaction, anxiety, anhedonia, and self-administration of drugs of abuse in intruders (Tornatzky and Miczek, 1994; Miczek et al., 2004; Rygula et al., 2005; Wood et al., 2010, 2012). Importantly, there is substantial individual variability in the magnitude of these consequences and in rats this has been associated with coping style (Krishnan et al., 2007; Wood et al., 2010). Repeated exposure of rats to resident–intruder stress results in the emergence of two distinct populations based on the latency to assume the subordinate defeat posture (Wood et al., 2010). The latency has a bimodal distribution, indicating that these are distinct populations rather than simply extremes of a normally distributed population. Rats that exhibit defeat with a relatively short latency (SL rats) show increased anhedonia, immobility in the forced swim test, decreased heart rate variability, and hypothalamic-pituitary-adrenal dysregulation similar to that reported in depression, compared to stressed rats that exhibit a long latency and resist defeat (LL rats; Wood et al., 2010, 2012,2015). Interestingly, the initial exposure to the stress results in relatively rapid onsets to defeat, whereas the LL phenotype develops with repeated exposures (Wood et al., 2010).

Given the decreased vulnerability of LL rats to rodent endpoints of stress-related psychiatric disorders, identifying neurobiological substrates and circuitry that distinguish the SL and LL phenotypes may reveal the basis for different stress coping strategies as well as vulnerability to stress-related pathology. In this regard, administration of CRF antagonists prior to repeated resident–intruder stress was demonstrated to bias coping strategy toward the LL phenotype and to prevent certain consequences of repeated resident–intruder stress, underscoring a potential role for CRF as a link between coping strategy and vulnerability to the pathologic consequences of social stress (Wood et al., 2012). This study used a functional neuroanatomical approach to determine whether afferent regulation of the LC differed between rats with different coping strategies to repeated stress. Retrograde tract tracing from the LC was combined with immunohistochemistry to detect c-fos, a marker of neuronal activation, and either CRF or ENK in LC afferents of rats exposed to repeated resident–intruder stress. To determine how this differed from rats exposed to an acute resident–intruder stress before the divergence of different coping strategies, similar approaches were applied to rats exposed to a single session of resident–intruder stress.

## Materials and Methods

### Experimental animals

All rats were ordered from Charles River Laboratories and housed in a controlled environment (20°C, 12 h light/dark cycle). Food and water were available *ad libitum*. Adult male Long–Evans retired breeder rats (650–850 g) were used as residents and adult male Sprague-Dawley rats (250–300 g) were used as intruders (or matched controls). Intruders were initially pair-housed. After the first experimental manipulation, they were housed individually. Residents were housed individually. Experiments were performed during the light cycle. All animal procedures were performed in accordance with the author’s Institutional Animal Care and Use Committee regulations.

### Fluorogold injection into the LC

Intruder rats and matched controls were anesthetized with a 2% isoflurane-air mixture, positioned in a stereotaxic instrument, and surgically prepared for electrophysiological localization of the LC using a glass micropipette. Micropipettes (15–20 μm diameter tip) were backfilled with a solution of 2% Fluorogold (FG; Fluorochrome) in 0.9% sterile saline. Microelectrode signals were led from a preamplifier to filters and additional amplifiers. Impulse activity was monitored on an oscilloscope and with a speaker to aid in localizing the LC. When neuronal activity characteristic of the LC was localized (spontaneous discharge rate of 1–4 Hz, entirely positive notched waveform of 2–3 ms duration in unfiltered trace and biphasic response to tail or paw pinch), the micropipette was repositioned until the core of the LC was located, after which FG was iontophoresed (5 μA, 7 s duty cycle, 15 min). The micropipette was then kept in place for 10 min to prevent leakage. A dense CRF terminal field exists in the dorsolateral peri-LC where CRF axon terminals synapse with LC dendrites. The major source of these terminals is the central nucleus of the amygdala, which does not project to the LC core (Aston-Jones et al., 1986; Van Bockstaele et al., 1998). To assure labeling of this major CRF afferent, after iontophoresing FG into the LC core the pipette was repositioned to the dorsolateral peri-LC for an additional 15 min application of FG followed by a 10 min post-iontophoretic period. The micropipette was removed, the scalp wound was sutured, and rats were allowed to recover 3 d before experimental manipulations.

### Social stress

Sprague-Dawley rats were randomly assigned to intruder (*n* = 7) or matched control (*n* = 7) groups. Intruder rats were placed into the cage of the resident. After a brief period of investigation, an aggressive encounter by the resident typically ensued. A mesh wire partition was placed between the intruder and resident either immediately after the intruder assumed a supine posture signaling subordination for 3 s (defeat) or after 15 min had elapsed from the time when the intruder was placed into the cage if no defeat occurred. The partition prevented further physical contact, but allowed for exposure to visual, auditory, and olfactory cues for the remainder of a 30 min session. For repeated social stress, this was repeated for 5 consecutive days, with intruders being exposed to a different resident on each day. The latency to assume the subordinate posture was recorded for each rat for each exposure to resident–intruder stress. The mean latency over the 5 d was calculated for each rat and analyzed by a cluster analysis across the group (JMP 9.0.0, SAS Institute; www.jmp.com). Control rats were placed in a novel cage for 30 min daily, with the final 15 min behind the wire partition. All rats were returned to their home cage following each session.

In a separate experiment rats (*n* = 8) were injected with FG in the LC as described above. They then received either a single exposure to resident–intruder stress (*n* = 4) or control manipulation (*n* = 4). Latency to defeat was recorded in the stressed rats as described above.

### Immunohistochemistry

Rats were anesthetized with 5% isofluorane and transcardially perfused with 2% heparinized saline, followed by 4% paraformaldehyde, 90 min after the last experimental manipulation. The brain was removed and postfixed in 4% formaldehyde overnight at 4°C and stored in sucrose solutions of 10% and 20% for 1 h each, followed by 30% sucrose for 48–72 h in 0.1 m phosphate buffer (PB) containing 0.1% sodium azide at 4°C. One side of the brain was notched to verify tissue orientation following sectioning. Frozen 40-µm-thick sections were cut in the coronal plane in a series of four using a freezing microtome and collected in 0.1 m PB. Every fourth section through the rostrocaudal extent of the LC was collected and processed for immunoperoxidase detection of the extent of the FG injection site. Likewise, serial coronal sections through the PGi and CNA were processed for immunoperoxidase visualization of FG to evaluate the magnitude of retrograde labeling. These sections were washed in 0.1 m Tris buffered saline (TBS), pH 7.6, and incubated in 0.5% bovine serum albumin (BSA) in TBS for 30 min. Subsequently, sections were incubated in 0.5% BSA and 0.25% Triton X-100 in 0.1 m TBS for 30 min and rinsed extensively in 0.1 m TBS. Sections were incubated in rabbit anti-FG (1:2,000; Chemicon International) for 15–18 h at room temperature. They were then rinsed and incubated in biotinylated donkey anti-rabbit (1:400; Jackson ImmunoResearch Laboratories) for 30 min followed by rinses in 0.1 m TBS. Subsequently, sections were incubated for 30 min in avidin-biotin complex (Vector Laboratories). FG was visualized by reaction with 3,3′-diaminobenzidine and 30% hydrogen peroxide in TBS. Sections were collected, dehydrated and coverslipped for light microscopic analysis of FG immunoreactivity.

A series of sections through the rostrocaudal segment of the PGi was processed for immunofluorescent visualization of FG, c-fos, and ENK and a series of sections through the CNA was processed for immunofluorescent visualization of FG, c-fos, and CRF. Only cases with the most restricted placement of FG and optimal retrograde labeling were used in the analysis. Free-floating sections were rinsed extensively in 0.1 m PB followed by rinses in 0.1 m TBS. Sections were then incubated in 0.5% BSA in 0.1 m TBS for 30 min, and rinsed in 0.1 m TBS. Following rinses, sections containing the PGi were incubated overnight at room temperature in a solution containing guinea pig anti-FG (1:2000; Protos Biotech), rabbit anti-c-fos (1:3000; Calbiochem) and mouse anti-ENK (1:100; Fitzgerald Laboratories) in 0.1 m TBS with 0.1% BSA and 0.25% Triton X-100. Likewise, tissue sections from the CNA were incubated overnight at room temperature in rabbit anti-FG (1:2,000; Chemicon International), mouse anti-c-fos (1:100; Santa Cruz Biotechnology) and guinea pig anti-CRF (1:2,000; Peninsula Laboratories). Sections were then washed in 0.1 m TBS and sections containing the PGi were incubated in a secondary antibody cocktail containing fluorescein isothiocyanate (FITC) donkey anti-rabbit (1:200; Jackson ImmunoResearch Laboratories), tetramethyl rhodamine isothiocyanate (TRITC) donkey anti-mouse (1:200; Jackson ImmunoResearch) and AlexaFluor 647 donkey anti-guinea pig (1:200; Jackson ImmunoResearch) antibodies prepared in 0.1% BSA and 0.25% Triton X-100 in 0.1 m TBS for 2 h in the dark. Sections from the CNA were incubated in a secondary antibody cocktail containing FITC donkey anti-mouse (1:200; Jackson ImmunoResearch Laboratories), TRITC donkey anti-guinea pig (1:200; Jackson ImmunoResearch), and AlexaFluor 647 donkey anti-rabbit (1:200; Jackson ImmunoResearch) antibodies. Following incubation with the secondary antibodies, the tissue sections were washed thoroughly in 0.1 m TBS, mounted on slides and allowed to dry in complete darkness. The slides were dehydrated in a series of alcohols, soaked in xylene and coverslipped using DPX (Sigma-Aldrich). Sections were visualized using an Olympus 1X81 laser microscope (Olympus) and images captured using Olympus Fluoview ASW FV1000 program (Olympus).

As an indication of stress-induced LC activation c-fos-immunoreactivity was also quantified in every fourth section through the LC. Free-floating sections were rinsed extensively in 0.1 m PB followed by rinses in 0.1 m TBS. Sections were then incubated in 0.5% BSA in 0.1 m TBS for 30 min, and rinsed in 0.1 m TBS for 10 min, three times. Following rinses, the sections were incubated overnight in a rabbit monoclonal antibody for c-fos (1:3000; Calbiochem) in 0.1% BSA and 0.25% Triton X-100 in 0.1 m TBS. Incubation time was 15–18 h in a rotary shaker at room temperature. The following day, sections were rinsed three times in 0.1 m TBS and incubated in biotinylated donkey anti-rabbit (1:400; Jackson ImmunoResearch Laboratories) for 30 min followed by rinses in 0.1 m TBS. A 30 min incubation of avidin-biotin complex (Vector Laboratories) followed. For all incubations and washes, sections were continuously agitated with a rotary shaker. C-fos was visualized by a 4 min reaction in 22 mg of 3,3′-diaminobenzidine (Sigma-Aldrich) and 10 µl of 30% hydrogen peroxide in 100 ml of 0.1 m TBS. A total of six sections were analyzed per rat and the mean number per group was obtained by determining the average c-fos-immunoreactive cells per section per rat. The mean number per rat was determined for comparison between groups.

### Data analysis

Sections from each rat were first examined for accurate and localized FG injections determined from the bright-field images (Olympus BX51). Images were captured using Spot Advanced software (Diagnostic Instruments). Only animals with satisfactory injection sites were used for data analysis. The criteria for defining satisfactory injection sites included the extent of diffusion of injections, proximity of injections to the target nucleus and retrograde labeling in the PGi and CNA. The area of the PGi that was analyzed extends from the anterior pole of the lateral reticular nucleus to the level of the caudal third of the facial nucleus, as previously described (Andrezik et al., 1981; Plates 62 to 68; Paxinos and Watson, 1998). The area of the CNA analyzed is located in the medial part of the amygdaloid complex which is bounded laterally by the basolateral amygdaloid nucleus, lateral amygdaloid nucleus, and amygdaloid intermedullary gray, medially by primary substantia innominata and medial amygdaloid nucleus, dorsally by the basal nucleus and interstitial nucleus of the posterior limb of the anterior commissure, and ventrally by the intercalated amygdaloid nucleus and intra-amygdaloid division of the bed nucleus of stria terminalis (Plates 25 through 30; Paxinos and Watson, 1998).

For quantification, every fourth coronal (120 μm apart) section was taken through the anteroposterior extent of the PGi and CNA. The number of c-fos, FG, ENK, or CRF, dually labeled cells (FG+c-fos) and triple-labeled cells (FG, c-fos, and ENK or CRF) were counted in 12 PGi-containing sections and six CNA-containing sections. Counts of c-fos in the LC were determined from six sections from each rat. The total number of labeled (FG, c-fos, Enk or CRF, FG/c-fos and FG/c-fos/ENK, or FG/c-fos/CRF) cells from all sections quantified was determined for each rat, as well as the percentage of FG-fos-labeled neurons that were triple-labeled and the group means were compared by a one-way ANOVA with Tukey *post hoc* tests for comparisons between individual groups (JMP 9.0.0, SAS Institute).

## Results

### Repeated stress and retrograde labeling

Repeatedly stressed rats clustered into two populations based on either a relatively SL or LL to assume the subordinate defeat posture, consistent with other reports (Wood et al., 2010). The range of latencies for the SL rats was 183–219 s with a mean latency of 196 ± 12 s (*n* = 3). The range of latencies for LL rats was 679–764 s with a mean latency of 730 ± 18 s (*n* = 4) and this was significantly different from the mean latency of SL rats (*p* = 0.005).

**Figure 1.**
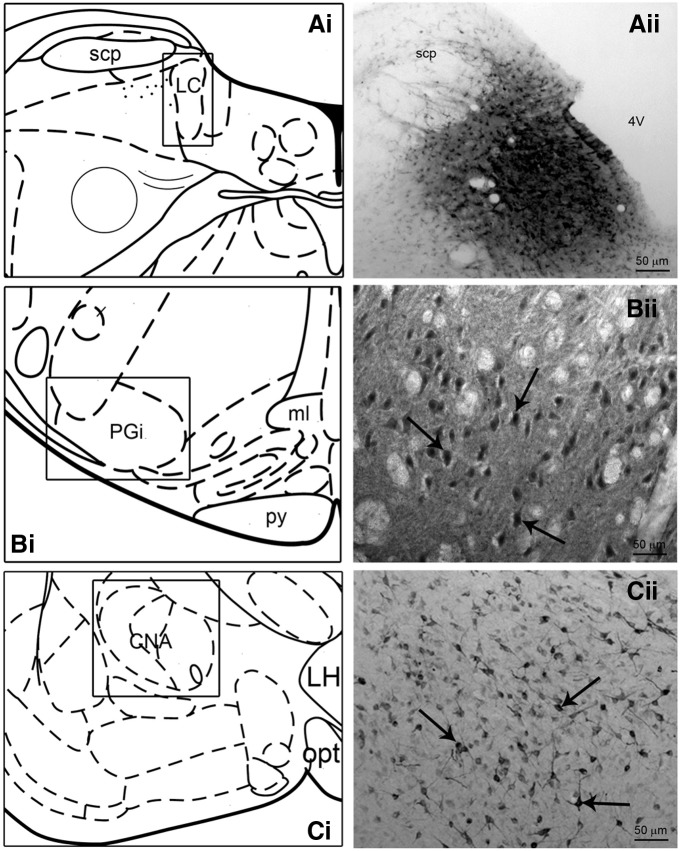
FG injection and retrograde labeling. ***Ai*–*Ci***, Schematic diagrams adapted from the Rat Brain Atlas (Paxinos and Watson, 1998) showing the anteroposterior levels of the representative injection site (***A***) and retrograde labeling (***B***,***C***). ***Aii***, Bright-field photomicrograph showing a representative FG injection within the rat LC. ***Bii*–*Cii***, Bright-field photomicrographs of representative retrograde labeling in the nucleus PGi (***Bii*** at ∼Plate 67; Paxinos and Watson, 1998) and CNA (***Cii*** at ∼Plate 26; Paxinos and Watson, 1998) following FG injection into the LC. The arrows indicate immunoperoxidase labeled cells. Scale bars: ***A***, 50 μm; ***B***, ***C***, 25 μm.

Of 14 rats (7 control, 7 stress) that were injected with FG into the LC 11 (5 control, 3 stress LL rats and 3 stress SL rats) had optimally placed injections that targeted the region of LC. Figure 1*A**ii* shows a representative bright-field photomicrograph depicting an FG injection site into the LC. FG injections into the LC yielded consistent retrograde labeling of perikarya in both the PGi and the CNA in all cases examined (Fig. 1*B*,*C*). The mean number of retrogradely labeled neurons after repeated social stress was not different between experimental groups in either the PGi (*F*_(2,8)_ = 2.034, *p* = 0.169) or CNA (*F*_(2,8)_ = 1.511, *p* = 0.278; Tables 1, 2).

**Table 1.** Repeated social stress, PGi

	**c-fos**	**FG**	**ENK**	**FG+c-fos**	**FG+c-fos, %**	**FG+c-fos expressing ENK, %**
Control	31 ± 3	129 ± 2	234 ± 7	22 ± 1	17 ± 1	9 ± 2
SL	31 ± 1	120 ± 4	256 ± 12	21 ± 1	18 ± 1	11 ± 3
LL	55 ± 4*^,#^	120 ± 4	286 ± 5*	40 ± 1*^,#^	33 ± 1*^,#^	41 ± 9**^,^ ^##^

**p < 0.01, *p < 0.05 *post hoc* comparisons to control values; ## *p* < 0.01, # *p* < 0.05 *post hoc* comparisons to SL values.

**Table 2.** Repeated social stress, CNA

**CNA**						
	**c-fos**	**FG**	**CRF**	**FG+c-fos**	**FG+c-fos, %**	**FG+c-fos expressing CRF, %**
Control	75 ± 4	240 ± 6	130 ± 5	17 ± 2	7 ± 1	12 ± 1
SL	160 ± 6*^,‡^	252 ± 10	212 ± 10*^,‡^	56 ± 4*^,‡^	22 ± 1*^,‡^	57 ± 5***,^‡ ‡^
LL	102 ± 12	212 ± 28	127 ± 16	27 ± 3	13 ± 1*	26 ± 5

***p < 0.001, *p < 0.05 *post hoc* comparisons to control values; ‡ ‡ *p* < 0.01, ‡ *p* < 0.05 *post hoc* comparisons to LL values.

### Repeated social stress-induced activation of LC-projecting ENK neurons in the PGi

**Figure 2.**
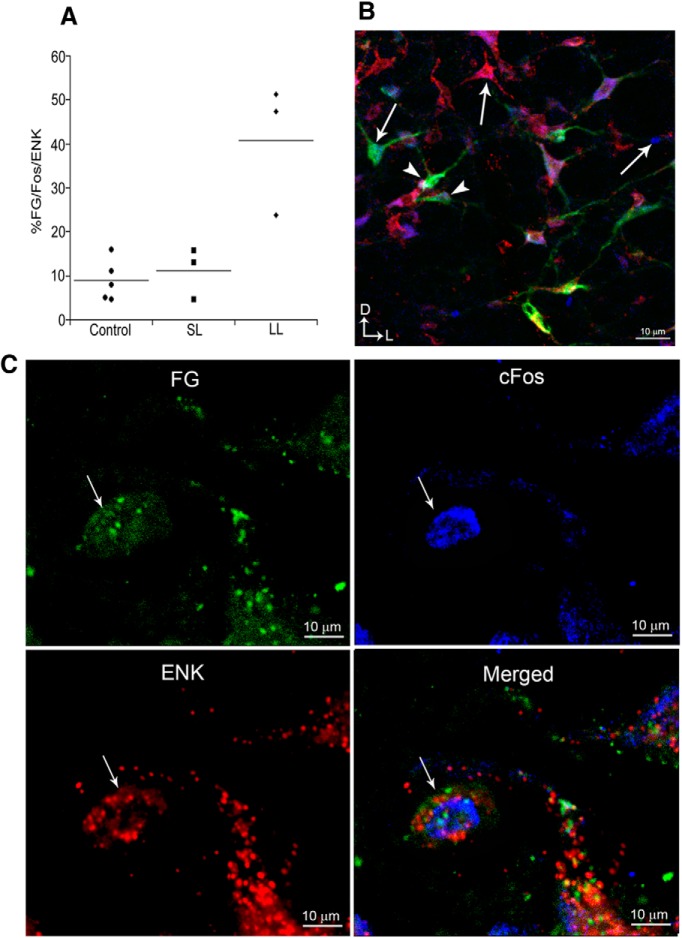
Activation of locus coeruleus-projecting enkephalin neurons in the nucleus PGi. ***A***, Scatterplot showing the percentage of FG-c-fos-labeled neurons that were also immunolabeled for ENK for individual control, SL, and LL rats. Lines through the points indicate the group mean. ***B***, Representative immunofluorescence photomicrograph from a long latency rat showing c-fos profiles (blue), FG labeling (green), ENK-immunoreactivity (red), and triple-labeled neurons (yellow). Arrows point to single-labeled neurons and arrowheads point to triple-labeled neurons. Scale bar, 10 μm. ***C***, High-magnification photomicrographs illustrating activated LC-projecting ENK neurons in the PGi of a LL rat. FG, c-fos, and ENK panels show single labeling for the retrograde tracer, FG (green), c-fos immunoreactivity (blue), and ENK immunoreactivity (red), respectively. The merged image shows all labels. Thin arrows point to the same triple-labeled neuron in all images. Scale bar, 10 μm.

Figure 2 shows examples of FG, c-fos, and ENK immunolabeling in the PGi. A clear distinction in PGi neuronal activation was observed between rats with different coping strategies after repeated exposure to social stress. More PGi neurons were activated in LL rats compared with control and SL rats as evidenced by the total number of c-fos profiles (*F*_(2,8)_ = 19, *p* = 0.0009; Table 1). Moreover, a greater number of PGi cells that project to the LC were activated in LL rats compared with control and SL rats (FG+c-fos; *F*_(2,8)_ = 52, *p* < 0.0001; Table 1). Interestingly, ENK-labeled neurons were also more abundant in LL rats compared with controls, but not SL rats (*F*_(2,8)_ = 7.59, *p* = 0.014; Table 1). Importantly, there was evidence of greater ENK-drive to the LC in LL rats compared with SL and control rats as indicated by a greater percentage of retrogradely labeled c-fos-expressing neurons that also were ENK-immunoreactive (*F*_(2,8)_ = 14, *p* = 0.002; Table 1).

### Repeated social stress-induced activation of LC-projecting CRF neurons in the CNA

**Figure 3.**
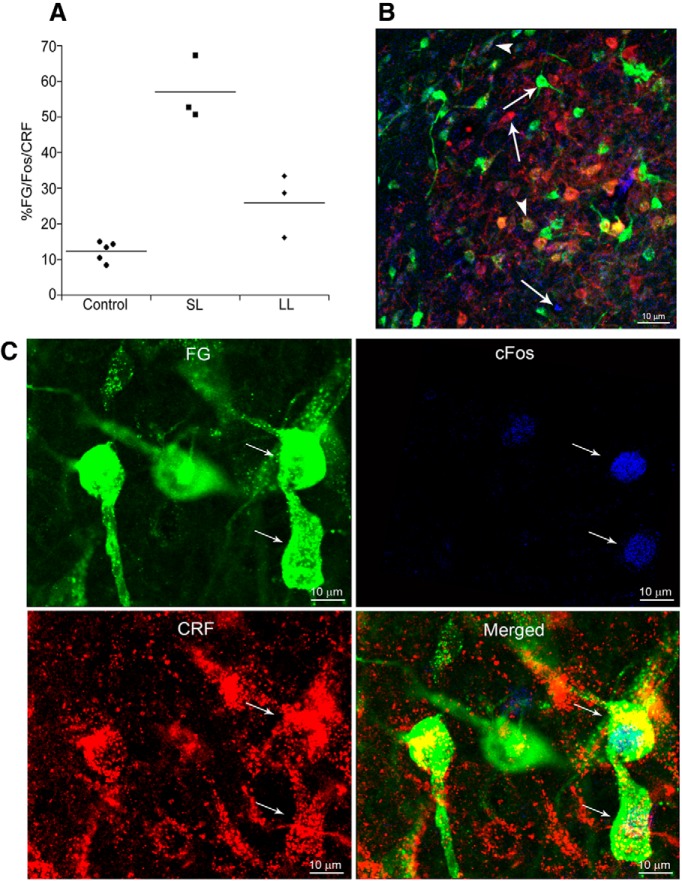
Activation of LC-projecting corticotropin-releasing factor neurons in the CNA. ***A***, Scatterplot showing the percentage of FG-c-fos-labeled neurons that were also immunolabeled for CRF for individual control, SL, and LL rats. Lines through the points indicate the group mean. ***B***, Representative immunofluorescence photomicrograph from a SL rat showing c-fos profiles (blue), FG labeling (green), CRF-immunoreactivity (red), and triple-labeled neurons (yellow). Arrows point to single-labeled neurons and arrowheads point to triple-labeled neurons. Scale bar, 10 μm. ***C***, High-magnification photomicrographs illustrating activated LC-projecting CRF neurons in the CNA of a SL rat. FG, c-fos, and CRF panels show single labeling for the retrograde tracer, FG (green), c-fos immunoreactivity (blue), and CRF immunoreactivity (red), respectively. The merged image shows all labels. Thin arrows point to the same triple-labeled neuron in all images. Scale bar, 10 μm.

Figure 3 shows examples of FG, c-fos, and CRF immunolabeling in the CNA. In direct contrast to the patterns of activation within the PGi, more CNA neurons overall were activated in SL rats compared with LL or control rats based on the number of c-fos profiles (*F*_(2,8)_ = 33, *p* = 0.0001; Table 2) and more CNA LC-afferents were activated in SL rats compared with other groups (FG+c-fos; *F*_(2,8)_ = 40, *p* < 0.0001; Table 2). Notably, CRF-immunolabeled neurons were more numerous in the CNA of SL rats compared with all other groups (*F*_(2,8)_ = 20, *p* = 0.0007; Table 2). Moreover, SL rats showed greater activation of CRF-LC-projecting neurons as indicated by the percentage of CRF-immunolabeled LC-projecting neurons exhibiting c-fos immunolabeling compared with both controls and LL rats (*F*_(2,8)_ = 43, *p* < 0.0001; Table 2). There was a tendency for more triple-labeled neurons in LL rats compared with controls (*p* = 0.052, Tukey's HSD).

### Acute social stress-induced activation of LC-projecting ENK neurons in the PGi and CRF neurons in the CNA

Consistent with previous reports that the LL phenotype emerges with repeated exposure to social stress (Wood et al., 2010), a group of rats that were exposed to a single session of resident–intruder stress all had short latencies to defeat ranging between 112 and 300 s with a mean of 205 ± 40 s (*n* = 4). As for repeatedly stressed rats, a single exposure to stress did not affect retrograde labeling from the LC and the numbers of FG-labeled neurons were similar in stressed and matched controls in both the PGi (*F*_(1,6)_ = 2.445, *p* = 0.169) and CNA (*F*_(1,6)_ = 1.206, *p* = 0.314; Tables 3,4). The single-stress exposure increased c-fos labeling compared with control manipulation in both the PGi (*F*_(1,6)_ = 14, *p* = 0.0009) and CNA (*F*_(1,6)_ = 86, *p* < 0.0001; Tables 3,4). Acute social stress was associated with increased ENK-immunolabeled cells in the PGi (*F*_(1,6)_ = 11.5, *p* = 0.015) and CRF-immunolabeled cells in the CNA (*F*_(1,6)_ = 54.5, *p* = 0.0003) compared with control manipulation (Tables 3,4). Importantly, rats acutely exposed to the social stress displayed evidence of both greater ENK and CRF drive to the LC from the PGi and CNA, respectively, compared with controls. The percentage of LC-projecting PGi neurons and CNA neurons that express c-fos and were also ENK- or CRF-immunoreactive, respectively, and was greater in stressed compared with control rats (*F*_(1,6)_ = 71, *p* < 0.0001 and *F*_(1,6)_ = 52, *p* < 0.0001, respectively; Tables 3,4).

**Table 3.** Acute social stress, PGi

	**c-fos**	**FG**	**ENK**	**FG+c-fos**	**FG+c-fos, %**	**FG+c-fos expressing ENK, %**
Control	37 ± 4	137 ± 5	224 ± 8	18 ± 3	13 ± 2	15 ± 4
Defeat	57 ± 4**	155 ± 12	255 ± 6*	12 ± 2	15 ± 2	52 ± 2***

***p < 0.001, **p < 0.01, *p < 0.05; one-way ANOVA comparisons to control values.

**Table 4.** Acute social stress, CNA

	**c-fos**	**FG**	**CRF**	**FG+c-fos**	**FG+c-fos, %**	**FG+c-fos expressing CRF, %**
Control	88 ± 4	220 ± 6	142 ± 7	20 ± 4	9 ± 2	19 ± 2
Defeat	180 ± 9***	237 ± 12	221 ± 9***	57 ± 2***	24 ± 2***	70 ± 7***

***p < 0.001; one-way ANOVA comparisons to control values.

**Table 5.** Statistical table

	**Data structure**	**Test type**	**Power**
a. Table 1-PGi	Normal distribution	ANOVA/Tukey	0.99
b. Table 2-CAN	Normal distribution	ANOVA/Tukey	0.99
c. Table 3-PGi	Normal distribution	ANOVA/Tukey	0.82
d. Table 4-CAN	Normal distribution	ANOVA/Tukey	1.00

### Acute and repeated social stress, and LC activation

**Figure 4:**
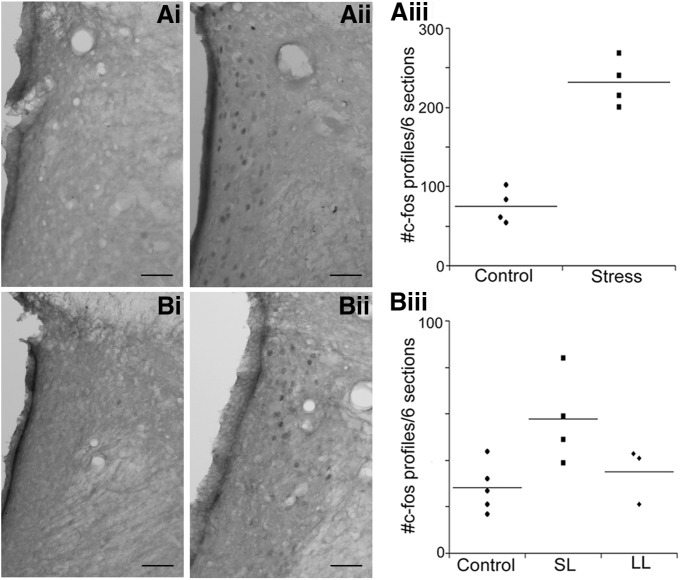
Activation of LC neurons by a single or repeated exposure to resident–intruder stress. ***Ai*, *Aii***, Representative sections from rats exposed to a single control manipulation (***Ai***) and a single resident–intruder stress (***Aii***). ***Aiii***, Scatterplot showing the number of c-fos profiles in the LC after a single stress or control manipulation for individual control and stressed rats. Lines through the points indicate the group mean. ***Bi***, ***Bii***, Representative sections from a control rat exposed to five repeated manipulations (***Bi***) and an SL rat exposed to five repeated resident–intruder exposures (***Bii***). ***Biii***, Scatterplot showing the number of c-fos profiles in the LC after the fifth stress or control manipulation for individual control, SL, and LL rats. Lines through the points indicate the group mean.

To determine whether differences in stress-induced activation of LC afferents have consequences for LC activity, c-fos-immunoreactive profiles were quantified in this region in the different groups. Figure 4 shows examples of c-fos-immunoreactive profiles in representative LC sections of rats exposed to a single or repeated resident–intruder stress or their matched controls. The number of c-fos-immunoreactive profiles in the LC was greater in acute stressed compared with control rats (*F*_(1,6)_ = 71, *p* = 0.0002; Fig. 4). Notably, after five repeated exposures c-fos-immunoreactive profiles in the LC were elevated above controls in SL, but not LL rats (*F*_(2,9)_ = 4.92, *p* = 0.036; Fig. 4).

## Discussion

Electrophysiologic studies demonstrated that during acute stress LC neurons are coregulated in an opposing manner by CRF-mediated excitation and opioid-mediated inhibition, with CRF excitation predominating (Curtis et al., 2001, 2012). The present neuroanatomical evidence that CNA-CRF and PGI-ENK afferents to the LC are both engaged during acute resident–intruder stress and that the net effect is LC activation is consistent with the electrophysiologic findings. Importantly, the current study demonstrated that with repeated social stress, the establishment of different coping strategies is associated with strategy-specific changes in the circuitry that regulates the brain norepinephrine system. Specifically, in rats with a tendency to resist subordination, the inhibitory ENK drive to the LC was maintained, whereas activation of CNA-CRF LC afferents was lost and LC activation was no longer apparent. In direct contrast, rats that show a propensity toward subordination exhibited the opposite pattern, with a prominent activation of excitatory CNA-CRF afferents and LC activation, but a loss of PGI-ENK drive. The results are consistent with the differential stress-induced internalization of μ-opioid receptors (MOR) in LC neurons of LL rats and CRF1 receptors in LC neurons of SL rats (Chaijale et al., 2013). Together, they suggest that repeated social stress engages distinct circuits that have opposing influences to regulate activity of the LC-NE system in rats with different coping strategies. This differential circuit activation may determine the coping strategy and/or the pathologic consequences of the stress.

### Technical considerations

Interpretations of the current findings must take into account certain caveats of retrograde tract tracing and immunolabeling of neuropeptides. The extent and localization of the FG injection cannot be identical across rats. Nonetheless, monitoring electrophysiologic characteristics during micropipette placement greatly optimizes the degree of accuracy of injections. The use of iontophoresis through small diameter micropipettes limits diffusion and reduces the degree of axonal damage and uptake by fibers of passage. Cases that are analyzed are conservatively limited to those in which the injection fills the LC and dorsolateral peri-LC where LC dendrites extend. Only cases with retrograde labeling in the major LC afferents, the nucleus PGi, and CNA are used. Confirming the consistency of retrograde labeling, there were no differences between groups in the number of retrogradely labeled neurons in the PGI or CNA. Importantly, this was also true when comparing acute and repeatedly stressed rats even though FG was present for a shorter duration in acutely stressed rats (4 d) compared with repeatedly stress rats (8 d). Another caveat is that greater visualization of the neuropeptides could be obtained with colchicine, which was not used in the present study because it affects c-fos expression as well as retrograde labeling (Gorenstein et al., 1985; Monti-Graziadei and Berkley, 1991). Because of this, the percentage of triple-labeled neurons could be underestimated in this study. Given that CRF and ENK neurons were readily visible in all analyzed cases, the lack of colchicine should not have a bearing on interpretations. Finally, c-fos profiles were observed in control rats although their number was substantially less compared with acutely stressed rats or repeatedly stressed SL rats. This was not unexpected as the controls were significantly manipulated. To mimic the handling of the stressed rats, controls were placed into a novel cage, picked up at 15 min, and put behind a wire mesh barrier that limited their access to the entire cage. This procedure assured that any differences between control and stressed groups were entirely due to the presence of the resident.

### Stress-induced coregulation of LC activity

Activation of LC neurons by acute stressors is an important central limb of the stress response. Converging lines of evidence implicate CRF as a neurotransmitter that mediates this response. CRF-immunoreactive axon terminals synapse with tyrosine hydroxylase-immunoreactive dendrites in the nucleus LC and peri-LC (Van Bockstaele et al., 1996, 1998). CRF increases LC neuronal discharge rates *in vivo* and in slice preparations *in vitro* (Curtis et al., 1997; Jedema and Grace, 2004). Importantly, local administration of CRF antagonists prevent LC activation by certain stressors (Valentino et al., 1991; Lechner et al., 1997; Kawahara et al., 2000). LC neurons are also regulated by endogenous opioids during acute stress. ENK axon terminals synapse with LC dendrites (Van Bockstaele et al., 1995). MOR agonists potently inhibit LC neurons *in vivo* and in slice preparations *in vitro* (Williams and North, 1984; Nestler et al., 1994). MOR regulation of LC activity is engaged during acute stress, as indicated by a naloxone-sensitive LC neuronal inhibition that is unmasked when stressed rats are administered a CRF antagonist (Curtis et al., 2001, 2012). Notably, there is little colocalization of CRF and ENK in axon terminals in the LC region and convergence of CRF and ENK axon terminals onto the same LC dendrites is more prominent (Tjoumakaris et al., 2003). Additionally, evidence suggests that these derive from distinct sources, with ENK deriving primarily from the PGi and CRF from the CNA (Drolet et al., 1992; Van Bockstaele et al., 1998; Tjoumakaris et al., 2003). Although the net effect of acute stress on LC neuronal discharge rate is CRF-mediated excitation, eliminating the excitatory CRF influence with an antagonist reveals a naloxone-sensitive, opioid-mediated inhibition (Curtis et al., 2001, 2012). The engagement of opioid afferents to the LC during acute stress serves to maintain LC activation in an optimal range and to facilitate a return to baseline discharge when the stressor is terminated. (Curtis et al., 2001, 2012). The present neuroanatomical findings in rats exposed to a single resident–intruder stress were consistent with the electrophysiologic results by providing evidence for activation of both ENK and CRF afferents to the LC and overall LC activation as indicated by c-fos expression.

### Role of coping strategy

Rats initially exposed to resident–intruder stress assume defeat with short latencies (Wood et al., 2010; the present study). With repeated exposure, two subpopulations emerge that are characterized by a propensity versus resistance to assume the subordinate defeat posture (Wood et al., 2010). Interestingly, it is the LL population that emerges and becomes significantly different by the fourth exposure (Wood et al., 2010). The two populations are also distinguished by the consequences of stress. For example, SL rats show evidence for hypothalamic-pituitary-adrenal axis dysfunction, anhedonia, immobility in the forced swim test and decreased heart rate variability (Wood et al., 2010, 2012,2015). As these are characteristics that are seen in major depression, the SL and LL phenotypes have been interpreted to represent stress-vulnerable and stress-resilient populations, respectively. This may be a simplistic interpretation as both coping strategies may be associated with distinct pathologies and at the same time have evolutionary adaptive purpose. Nonetheless, it is important to identify the neurobiological basis and neurocircuitry underlying the different coping strategies.

A comparison of CRF1 and MOR cellular localization following a bout of repeated resident–intruder stress suggested that ENK and CRF were differentially engaged in rats with different coping strategies to repeated social stress (Chaijale et al., 2013). Thus, MOR was internalized in LC dendrites selectively in LL rats, whereas CRF1 was selectively internalized in SL rats. As receptor internalization is a consequence of agonist binding, these findings suggest that ENK is preferentially released to interact with MOR in LL rats and CRF released to interact with CRF1 in SL rats.

**Figure 5.**
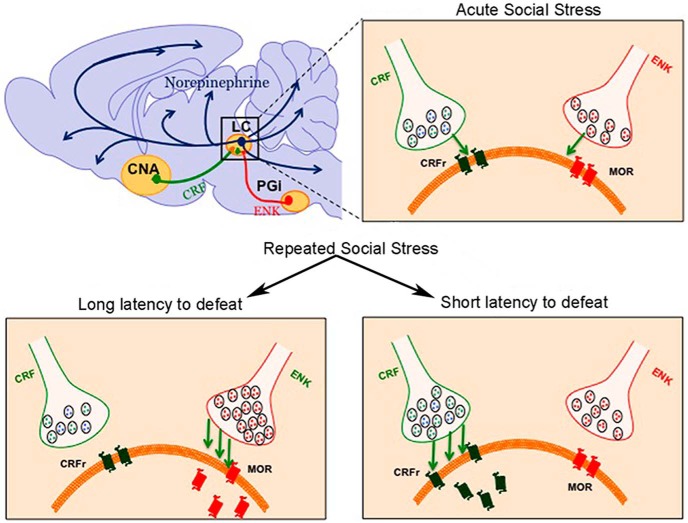
Schematic depicting distinct engagement of CRF and ENK afferents to the LC and adaptations in receptors in LC neurons depending on social stress history and coping strategy. Acute social stress engages CRF inputs to the LC from the CNA and ENK inputs from the PGi to the LC. The emergence of different coping strategies with repeated social stress is associated with distinct biases toward regulation by one afferent. In rats that resist defeat (LL rats) PGi-ENK afferents to the LC remain engaged resulting in MOR internalization and upregulation in this phenotype. The CRF influence is decreased in LL rats. In contrast, in rats exhibiting a subordinate coping style (SL), amygdalar-CRF afferents are engaged and CRF1 becomes internalized in LC dendrites. The PGi-ENK influence is diminished in these rats. The selective engagement of afferents with opposing effects that are related to coping styles may be a basis for individual differences in the pathological consequences of social stress.

The present findings using functional neuroanatomy support the interpretations of the electron microscopic results (Fig. 5). In LL rats, PGi-ENK afferents to the LC were activated after the fifth social stress and this was not apparent in SL rats. Rather, in SL rats, CNA-CRF inputs to the LC were activated. The net effect was selective activation of LC neurons in SL rats. Although the neuroanatomical results cannot be analyzed across days in the same rats, an examination of the results across days suggests that as the LL coping style emerges, the ENK drive is maintained, whereas the CRF drive is diminished in LL rats resulting in a loss of LC activation. In SL rats, the CRF drive is maintained and the ENK drive is lost so that LC activation remains. Taken with findings that administering a CRF antagonist prior to each resident–intruder stress biases the coping style toward LL, the results suggest that CRF promotes the SL coping strategy (Wood et al., 2012). Notably, in all cases in which triple-labeled neurons increased in the PGI or CNA, the number of neurons expressing either ENK or CRF was also greater in the respective group, suggesting that the repeated stress may be affecting neurotransmitter expression in LC afferents that are activated during the stress.

### Functional and clinical implications

Given that CRF1 and MOR activation regulate the LC in opposing manners, the differential engagement of CRF and ENK afferents in rats with different coping styles has important implications Figure 5. One question is whether there is a causal link between afferent regulation of the LC and coping strategy. The finding that systemic administration of a CRF antagonist before each exposure to resident–intruder stress shifts the coping strategy phenotype toward LL, supports causality between CRF and the SL strategy (Wood et al., 2012). It is also possible that coping strategy determines the afferent regulation that is engaged. Alternatively, the circuitry may be designed such that the afferents engaged to modulate LC activity and the circuitry determining the coping strategy are regulated in parallel by an upstream circuit component.

Afferent regulation of LC activity would be predicted to impact the behavioral consequences of stress. The “depressive-like” behavioral phenotype of SL rats is attenuated in rats pretreated with a CRF antagonist and this treatment biases the coping strategy toward LL (Wood et al., 2012). It is tempting to speculate that this is, in part, a result of CRF actions at the level of the LC. Notably, rats with intracranial implants for LC recordings exhibit an LL phenotype response to repeated social stress and show electrophysiologic evidence for opioid regulation of the LC (Chaijale et al., 2013). In these rats, naloxone produces an activation of LC neurons that resembles that seen during opioid withdrawal. These findings have suggested that repeated social stress produces a state of mild opioid dependence in rats with the LL coping strategy that could predispose to substance abuse. Together, the present results suggest that different stress-coping strategies are associated with distinct circuitry that can regulate the LC-norepinephrine system in opposing manners and result in pathology that is unique to the specific coping phenotype.

## Synthesis

The decision was a result of the Reviewing Editor Quentin Pittman and the peer reviewers coming together and discussing their recommendations until a consensus was reached. A fact-based synthesis statement explaining their decision and outlining what is needed to prepare a revision is listed below. The following reviewer(s) agreed to reveal their identity: Cheryl Sisk

The reviewers and I have had considerable discussion over some issues raised in the second review.

One issues was that the series of experiments in which a single stress was applied allowed only 3 days for FG to accumulate and thus might not be adequate to allow sufficient time for the retrograde transport to take place and thus give you a true quantitative picture of FG labeled cell numbers. However, it was noted that there seemed to be similar numbers of cells labeled after both the single and the repeated stress, indicating that both 3 and 8 days of retrograde transport gave similar numbers of cells labeled. You comments on this in rebuttal would be appreciated.

A second point was a continuing concern that injecting in the peri-nuclear area of the LC may label a different population of afferents than does application of the FG only into the LC proper. Please address this in your rebuttal and perhaps acknowledge this factor in the discussion.

Reviewer's comments:

The authors have been somewhat responsive to the concerns raised by the reviewers, although key problems that were noted in the previous review were addressed by argument instead of data collection, and there are several major deficiencies in the experimental design and interpretation thereof.

The central confound that remains in this study concerns the lack of specificity in retrograde labeling. The referee appreciates that LC dendrites extend outside of their cytoarchitectonic boundary; nevertheless, virtually no one doing tracer injection experiments (iontophoretic or otherwise) needs to ever "worry" about a tracer not diffusing out of such a small structure like LC. Spread of tracer outside of the target region is a fact of anatomical tracing using conventional approaches in structures in the rat brain stem. It is a shame given that the authors do such an elegant job of identifying LC electrophysiologically, administer tracer iontophoretically, that they then turn around and hit an adjacent region in the same surgery. Without anatomical specificity, the authors are greatly hampered in their ability to make any specific inferences about circuitry.

The other lingering issue concerns the fact that Fos expression levels are high in control rats. The explanation provided by the authors is not convincing. Animals should habituate to repeated handling, and novel cage exposure should not be novel after several days. Neverthless, levels in controls remain roughly equivalent when one compares numbers across acute and repeated experiments. Moreover, the fact that Fos detection method used in this study is different (i.e., immunofluorescence versus peroxidase labeling in other work) provides no basis for the explanation that Fos levels in controls remain elevated. Finally, the idea that the tracer injection surgery may have influenced Fos levels would not be expected to be a problem (it is false that few studies have ever examined Fos after a surgical manipulation or tracer injection). One thing that did catch my attention was that behavioral procedures were conducted only 3 days after tracer injections. This is a very short recovery period, particularly to allow sufficient time for retrograde transport to CNA. While this was somewhat compensated for by the fact that stress exposure occurred over a 5-day period, a 3-day survival period in the acute stress studies is too brief to allow sufficient time for maximal retrograde labeling. While I am willing to concede the point about low numbers of doubly-labeled neurons reported, such a short survival time after surgery (i.e., 3 and 8 days for acute and repeated stress, respectively) raises the possibility that retrograde labeling, and hence colocalization with Fos, could have been much higher.

The authors have adequately addressed my concerns. The inclusion of data after a single social stress experience and the scatterplots are nice additions that strengthen the manuscript considerably.
